# Effect of working place infection control practices on workers' psychological distress: A large-scale cohort study during the COVID-19 pandemic in Japan

**DOI:** 10.3389/fpsyt.2022.933556

**Published:** 2022-09-16

**Authors:** Toyohiko Kodama, Tomohiro Ishimaru, Seiichiro Tateishi, Ayako Hino, Mayumi Tsuji, Akira Ogami, Tomohisa Nagata, Shinya Matsuda, Yoshihisa Fujino

**Affiliations:** ^1^Department of Nursing, School of Health Sciences, University of Occupational and Environmental Health, Kitakyushu, Japan; ^2^Department of Environmental Epidemiology, Institute of Industrial Ecological Sciences, University of Occupational and Environmental Health, Kitakyushu, Japan; ^3^Department of Occupational Medicine, School of Medicine, University of Occupational and Environmental Health, Kitakyushu, Japan; ^4^Department of Mental Health, Institute of Industrial Ecological Sciences, University of Occupational and Environmental Health, Kitakyushu, Japan; ^5^Department of Environmental Health, School of Medicine, University of Occupational and Environmental Health, Kitakyushu, Japan; ^6^Department of Work Systems and Health, Institute of Industrial Ecological Sciences, University of Occupational and Environmental Health, Kitakyushu, Japan; ^7^Department of Public Health, School of Medicine, University of Occupational and Environmental Health, Kitakyushu, Japan

**Keywords:** COVID-19, infection control, Kessler Psychological Distress Scale (K6), psychological distress, working place

## Abstract

**Background:**

The COVID-19 pandemic has dramatically transformed the work environment and practices worldwide. Long-term infection control practices may increase the psychological distress of workers, and, conversely, inadequate infection control practices in the working place may increase the fear of infection. This study aimed to determine the relationship between infection control practices in the working place and employee mental state during the COVID-19 pandemic in Japan.

**Methods:**

This study was conducted in December 2020 and February 2021. The participants had undergone a preliminary survey, which revealed that they were in a good mental state. Their psychological distress was investigated *via* a second survey, and the factors associated with distress were studied using a logistic model.

**Results:**

The results of the second survey indicated that 15.3% of participants demonstrated psychological distress. This was associated with leave-of-absence instructions, instructions for shortening business hours, and requests to avoid the working place in case of any symptoms.

**Conclusion:**

The study found that while some infection control practices reduce workers' distress, others worsen it. Employers need to consider infection control practices as well as the worsening mental state of employees following a decrease in income caused by such measures. Follow-up studies may be necessary to clarify the long-term effects on workers' mental states.

## Introduction

The COVID-19 pandemic has brought about significant changes in public health, particularly in mental health. Fear of infection, unstable employment and economic conditions, as well as countermeasures against infection, such as avoidance of physical contact and restrictions on movement, have reduced opportunities for social interaction; this has had a deteriorating effect on the mental state of the population. Previous studies showed increased anxiety and mental burden in areas where lockdowns have been ordered (1). Other negative effects associated with lockdowns include worsening of mental illnesses, depression, alcohol dependency, and suicide (2–4). Along with healthcare, the COVID-19 pandemic has also dramatically transformed the work environment and practices (5–7). Various measures were implemented to prevent the COVID-19 infection in the working place, including mask-wearing, physical distancing, daily health checks, personal hygiene such as hand hygiene, and working from home. The implementation of appropriate infection control practices in the working place may positively affect the mental state of workers by creating a safe environment, which has been reported to reduce anxiety and depression (8, 9). Proactive infection control practices may not only reduce workers' anxiety and fear of infection but also increase their confidence in the working place. However, many infection control practices are efforts to maintain physical distance and reduce social contact, which have been associated with loneliness and psychological distress (10, 11). In the COVID-19 pandemic, other factors can also possibly cause psychological distress among workers. For example, in working place where telecommuting is difficult, such as restaurants and leisure facilities, shortening work hours or reducing work days to prevent infection may decrease workers' income. Low income is associated with poorer mental health (12, 13). Excessive infection control practices may also reduce workers' willingness to express their thoughts and feelings, reducing psychological safety in the working place. However, the factors contributing to the workers' psychological distress in working place infection control practices have not yet been clarified.

A previous study has shown that the mental state of the Japanese deteriorated during the early stages of the COVID-19 pandemic (12). This study by Kikuchi et al. was a longitudinal survey of Japanese mental states from February 2020 to April 2020 (12). However, the number of people infected during that period was about one-tenth of the number during the peak period, which has led to a gap in existing research. Additionally, no studies about workers' mental state were conducted during the peak of the outbreak in Japan, which experienced a rapid spread of the infection from January 2020. For instance, the third wave of infection struck Japan in December 2021, leaving over 7,000 people infected daily. However, as far as we know, no cohort studies have surveyed workers' mental states after the third wave. An increase in the number of infected people would have a serious impact on employment and the economy, forcing workers to take long-term measures to prevent infectious diseases in their working place. While long-term infection control practices may increase the psychological distress of workers, inadequate infection control practices in the working place may increase the fear of infection.

We hypothesized two hypotheses in this study: first, working place infection control practices would reduce psychological distress if they created a safe environment; second, if working place infection control practices continued to maintain physical distance and reduced social contact, workers' psychological distress would worsen. These two hypotheses were tested.

## Materials and methods

We conducted a prospective cohort study using an online questionnaire that focused on Japanese workers during the pandemic. The survey was commissioned by Cross Marketing Inc., (Tokyo, Japan). Of the registered monitors, 605,381 were sent an invitation *via* e-mail to participate. The sampling plan was designed to collect an equal number of respondents with comparable sex and office and non-office worker status. Of these, a total of 55,045 registered monitors answered the initial screening questions to participate in the survey, and 33,302 who matched the survey's criteria (worker status, region, sex, and age) responded to the survey (14). The baseline survey was conducted from December 22 to 26, 2020, in Japan, during the beginning of the third wave of the pandemic. We have already reported details from the Protocol for our study (14). Research data were gathered from participants who had employment contracts at the time of this study. The participants' data were allocated by sex, prefecture, and occupation. We were able to detect incorrect responses using several algorithms. First, we prepared a step-by-step question in which respondents were asked to choose the third highest number from a list of five numbers. A total of 93% of respondents gave the correct answer to this question. Second, the system recorded the time taken to answer the question. Third, responses from respondents who were extremely underweight or short in stature were judged to be incorrect. As the height and weight questions required numerical input using the keyboard, it was assumed that incorrect responses were more likely to occur than when the inputs were simple click responses. Many of the incorrect entries for height and weight were found to include “000” or “999.” Based on the statistical distribution of height among Japanese adults, we excluded values of 140 centimeters or less, as these are extremely exceptional. Fourth, we verified whether there were any inconsistencies in the responses to questions that were repeated throughout the survey. The questions used to check for inconsistencies were those that asked about the presence or absence of family members living with the respondent and the area of residence; of the 33,087 respondents, 27,036 were determined to have answered the questions appropriately. In particular, the question about the status of family living together was asked more than once; for example, “Do you have a roommate?” “Do you live with an elderly person?” and “Do you have pre-school children?” Respondents with discrepancies in their responses were excluded. In addition, those who were determined to have given incorrect answers in any of the above four conditions were often observed to have given incorrect answers in the other three conditions as well (14). As a result, from the initial 33,302 participants, only 27,036 were included in this study. After the baseline survey, we followed the cohort and conducted a follow-up survey from February.

This study was approved by the Ethics Committee of the University of Occupational and Environmental Health, Japan (R2-079 and R3-006).

### Assessment of workers' psychological distress

To assess workers' psychological distress, we used the Japanese version of the Kessler Psychological Distress Scale (K6) (15–17) at baseline and the follow-up survey. The validity of the Japanese version of the K6 was confirmed (16, 17). A follow-up study was conducted from February 18 to February 19, 2021. In the current study, the cutoff for psychological distress was a K6 score of five or higher. The validity of the cutoff scores has also been confirmed (17).

### Infection control against COVID-19 at the working place

We investigated the status of infection control against COVID-19 in the participants' working place in the baseline and follow-up study. We examined the presence of instructions from the working place regarding infection control following the re-declaration of the state of emergency in January 2021. The survey items about infection control in the working place covered leave-of-absence instructions, instructions for shortening business hours, limits to business travel, prohibitions against eating together, instructions for wearing a mask, instructions to disinfect thoroughly with alcohol when entering and leaving rooms, recommendations for daily temperature checks, encouragement of telecommuting, and requests not to come to work if not feeling well.

### Other covariates

We obtained information on participants' profiles, characteristics, and socioeconomic status of the company they worked at in the baseline survey. The follow-up survey items, which are thought to influence psychological distress, contained the following factors: sex, age, marital status, number of employees, job type [mainly desk work (e.g., clerical job, computer work), jobs mainly involving interpersonal communication (e.g., hospitality practice, sales position), and mainly labor (e.g., field operation, care staff)], and education.

### Statistics

**Figure 1:**
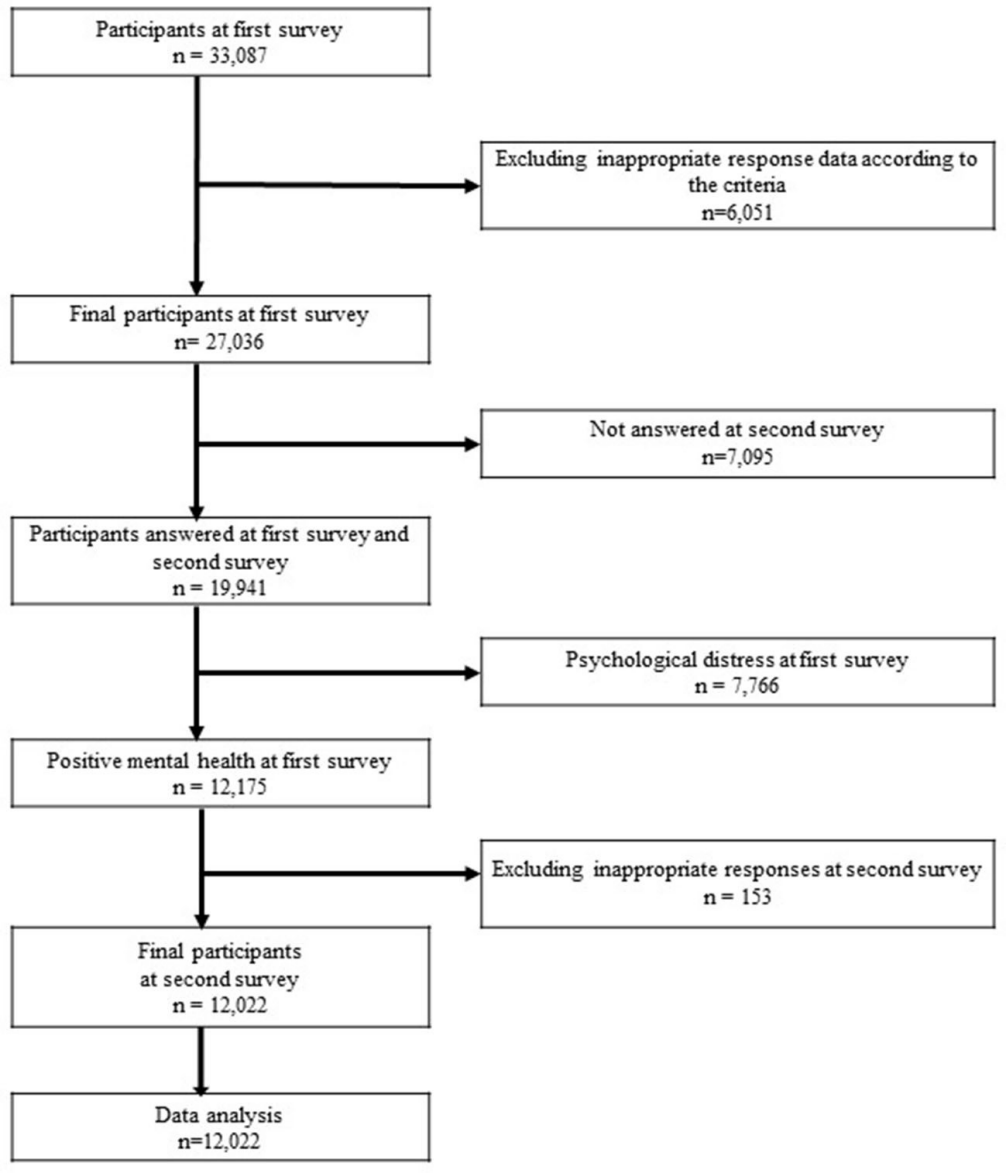
Flow diagram of the study.

To estimate the impact of the state of emergency declaration on infection control measures at the working place by examining depressed workers in the second survey, even though they were not psychologically distressed in the first survey. In the baseline survey, 7,766 participants who had a K6 score of five or higher were excluded, as our study focused on workers who had demonstrated robust mental state at baseline but then deteriorated, as evidenced in the follow-up survey. After excluding inappropriate responses and workers who were unemployed at the follow-up survey and adding those who reported a healthy mental state in the baseline survey, 12,022 workers were included in the analysis. This was followed by an analysis of the changes in the mental state of the participants, which were evidenced by the follow-up survey responses (see a flow diagram of the study in Figure 1).

Odds ratios (ORs) for psychological distress and instructions from working place regarding infection control were estimated using a logistic model. ORs were calculated by introducing all the instructions at the same time. Psychological distress was defined as a K6 score of five or higher. The multivariate model was adjusted for age, sex, marital status, number of employees, job type, and education. Working place measures to curb infection at the baseline involved the following: leave-of-absence instructions, instructions for shortening business hours, limits to business travel, prohibitions against eating together, instructions for wearing a mask, instructions to disinfect thoroughly with alcohol when entering and leaving rooms, recommendations for daily temperature checks, encouragement of telecommuting, and requests not to come to work if not feeling well. A *p*-value of < 0.05 was considered statistically significant. We used SPSS ver. 22 for Windows (IBM Corp., Tokyo, Japan) for analysis.

## Results

The follow-up survey found that, of the 12,022 participants, 1,842 (15.3%) exhibited psychological distress. Table 1 shows the characteristics of the participants whose responses were recorded regarding the number of infection control practices (age, K6 score, sex, marital status, job type, education). The average age was 49.6, the average score of K6 was 1.95, and more than half of the participants were married. Most workers in working places were <30 employees. The most common job type was “mainly desk work.” More than 70% of the participants reported that their educational background was that of vocational school.

**Table 1:** Characteristics of the participants according to the number of working place COVID-19 infection control practices.

	**Number of working**
	**place COVID-19**
	**infection control**
	**practices (*n* = 12,022)**
	Mean (SD) or %
Age (SD)	49.6 (9.9)
K6 (SD)	1.95 (3.7)
Sex, female	4,796 (39.9%)
Marital status	
Married	7,333 (61.0%)
Divorced or deceased spouse	1,137 (9.5%)
Unmarried	3,552 (29.5%)
Number of employees in the working place	
1–29	4,150 (34.5%)
30–99	1,742 (14.5%)
100–999	3,066 (25.5%)
≥1000	3,064 (25.5%)
Job Type	
Mainly desk work	6,494 (54.0%)
Jobs mainly involving interpersonal communication	2,803 (23.3%)
Mainly labor	2,725 (22.7%)
Education	
Junior high school	136 (1.1%)
High school	3,066 (25.5%)
Vocational school/college, university, graduate school	8,820 (73.4%)

Table 2 shows the number of implemented infection control practices in the working place and the details thereof. “Instructions for wearing a mask” (66.7%) was the most common infection control practice, followed by “thoroughly disinfect with alcohol when entering and leaving rooms” (64.0%). In contrast, the least common infection control practices were “instructions for leave of absence” (9.1%), followed by “instructions for shortening business hours” (10.2%).

**Table 2:** Implemented COVID-19 infection control practices in the working place.

	**Number of working**
	**place COVID-19**
Instructions for leave of absence	1,090 (9.1%)
Instructions for shortening business hours	1,228 (10.2%)
Refrain from or limit business travel	5,037 (41.9%)
Refrain from eating together	6,918 (57.5%)
Instructions for wearing a mask	8,016 (66.7%)
Thoroughly disinfect with alcohol when entering and leaving rooms	7,698 (64.0%)
Recommendations for daily temperature check	6,928 (57.6%)
Encouragement of telecommuting	3,150 (26.2%)
Request not to come to work when you are not feeling well	7,685 (63.9%)

Table 3 uses the logistic model to show the association between workers' distress and instructions from the working place regarding infection control. The multivariate model included age, sex, marital status, job type, and education. Psychological distress was strongly associated with instructions for leave of absence, instructions for shortening of business hours, and requests regarding not coming to work if unwell.

**Table 3:** Association between psychological distress and instructions from the working place regarding infection control.

	**Univariate**				**Multivariate** ^*^			
	**OR**	**95% CI**		***p***	**OR**	**95% CI**		***p***
**Instructions for leave of absence**	
Yes	reference				reference			
No	0.65	0.54	0.78	<0.001	0.66	0.55	0.79	<0.001
I do not know	0.95	0.54	1.67	0.864	0.94	0.53	1.65	0.821
**Instructions for shortening business hours**	
Yes	reference				reference			
No	0.79	0.66	0.94	0.009	0.78	0.65	0.94	0.008
I do not know	0.89	0.52	1.54	0.683	0.87	0.50	1.50	0.608
**Refrain from or limit business travel**	
Yes	reference				reference			
No	0.99	0.84	1.16	0.887	0.97	0.83	1.14	0.734
I do not know	1.31	0.93	1.85	0.120	1.25	0.88	1.76	0.207
**Refrain from eating together**	
Yes	reference				reference			
No	1.09	0.92	1.31	0.324	1.10	0.92	1.32	0.277
I do not know	1.14	0.77	1.71	0.514	1.12	0.75	1.67	0.583
**Instructions for wearing a mask**	
Yes	reference				reference			
No	0.98	0.81	1.19	0.828	1.02	0.84	1.24	0.858
I do not know	1.45	0.89	2.38	0.136	1.48	0.91	2.41	0.118
**Thoroughly disinfect with alcohol when entering and leaving rooms**.	
Yes	reference				reference			
No	1.06	0.88	1.28	0.514	1.06	0.88	1.28	0.546
I do not know	0.98	0.63	1.53	0.926	0.94	0.60	1.47	0.791
**Recommendations for daily temperature check**	
Yes	reference				reference			
No	0.88	0.75	1.03	0.111	0.93	0.80	1.10	0.400
I do not know	1.04	0.69	1.56	0.871	1.12	0.74	1.69	0.585
**Encouragement of telecommuting**	
Yes	reference				reference			
No	0.93	0.80	1.07	0.295	0.88	0.76	1.03	0.103
I do not know	1.06	0.73	1.54	0.754	1.03	0.71	1.50	0.876
**Request not to come to work when you are not feeling well**	
Yes	reference				reference			
No	1.28	1.07	1.52	0.008	1.31	1.09	1.56	0.003
I do not know	1.40	0.95	2.05	0.086	1.46	1.00	2.13	0.052

* The multivariate model included sex, age, marital status, number of employees, job type and education.

Participants who answered “No” to the question about instructions for leave of absence had significantly lower ORs (OR = 0.66, 95% CI = 0.55–0.79, *p* <0.00). Participants who answered “No” to the questions about instructions for shortening the number of business hours had significantly lower ORs (OR = 0.78, 95% CI = 0.65–0.94, *p* = 0.008). Participants who answered “No” to requests not to come to work if they were unwell had significantly higher ORs (OR = 1.31, 95% CI = 1.09–1.56, *p* = 0.003).

## Discussion

We examined the COVID-19 infection control practices in the working place during the re-declaration of the state of emergency and observed that, while some control practices had a significant favorable impact on workers' mental state, others had an unfavorable impact. In addition, workers in working place with little or no infection control practices were at a higher risk of psychological distress than workers in places with more infection control practices (other than instructions for leave of absence and shortening business hours).

This study showed that requests to “not come to work if not feeling well” were associated with a reduced risk of psychological distress. These results support our first hypothesis (working place infection control practices would reduce psychological distress if they created a safe environment). The absence of workers with poor health provides other workers with a sense of security that the infection will not be spread in the working place. Such measures also allow the workers who are feeling unwell to avoid the anxiety of infecting others. Sickness presenteeism is the act of going to work despite poor health; this has been observed prior to the COVID-19 pandemic. The reasons behind such behavior, including having a low income, unstable employment, guilt over increased burden on colleagues, and a lack of employees (18). Sickness presenteeism is known to be associated with poor mental health among workers (19). Workers who engage in frequent sickness presenteeism are reported to have a higher risk of developing depression in the future (20). The reasons are thought to include a worsening relationship with superiors and colleagues due to decreased work efficiency and poor sleep (20). On the contrary, during the COVID-19 pandemic, workers will not feel conflicted about taking a leave of absence if the working place has a clear policy of requesting not to come to work if they are not feeling well. In addition, reducing infection anxiety in the working place will help prevent the deterioration of workers' mental state. The company's proactive infection control practices may increase workers' confidence in the working place, leading to their psychological safety (21). Psychological safety is defined as individuals' perceptions of the consequences of taking interpersonal risks in their working place (22), and it has been shown to improve work performance, information sharing, and learning in the working place (23). In addition to the above, it has also been reported to be useful in preventing the deterioration of workers' mental health during the COVID-19 pandemic (24)—a finding that is consistent with our view.

The current study did not support our second hypothesis (i.e., if working place infection control practices continued to maintain that workers should physically distance themselves and reduce social contact, workers' psychological distress would worsen). However, if working place infection control practices continued to be implemented over an extended period of time, the results could be consistent with our second hypothesis. For example, refraining from eating together would decrease the risk of infection and reduce the fear of infection, but if this practice is prolonged, loneliness could be exacerbated by reduced communication and social interactions. Even if an infection control measure has a positive impact on mental health at one point in time, it may have different long-term effects.

Nevertheless, the instructions regarding leave of absence and shortening the business hours were associated with worsening workers' distress. Perhaps the workers' income decreased, and their economic situation worsened due to the instructions for leaves of absence and shortening business hours. Economic stress can affect a worsening mental state (25), and low income is also associated with poorer mental health (12, 13). As the leave of absence and shortening of business hours directly affect the worker's economic situation, it may have led to increased psychological distress. The second survey of this study was conducted from February 18 to 19, 2021; prior to that, a state of emergency was re-declared from January 8, 2021. In many areas, restrictions were placed on the hours of operation of restaurants, amusement centers, and other establishments that attract large numbers of people, as well as on serving alcoholic beverages. As workers in these occupations are often part-timers or non-regularly employed (26), who have lower incomes than those in regular employment (27), the decrease in income may have had a significant impact on psychological distress.

This study suggests that infection control practices in the working place are expected to reduce the prevalence of COVID-19 infections and are also beneficial to the workers' mental health. In the COVID-19 pandemic, as mental health is an emergent public health issue, infection control in the working place should be encouraged, as well as infection prevention and mental health support. Requests to not come to work when employees are not feeling well, which have been effective for workers' mental health, have been implemented in more than 60% of working places, but increased implementation is desirable. On the other hand, infection control practices that lead to a decrease in income were associated with worsening psychological distress, suggesting the need for employers to consider not only infection control practices but also worsening mental health. It would be advisable to make careful decisions regarding instructions for leave of absence and shortening business hours and to provide financial support as well. Naturally, infection control measures will be implemented differently depending on the type of work. For example, the infection control practices implemented in the food and medical service industries, which require on-site labor, will differ from those in industries where workers can easily shift to work at home. Even within the same type of work, managers and frontline workers may be affected differently by infection control practices in the working place. Organizational culture may also influence the willingness to take infection control measures in the working place and the mental state of workers; however, this study did not go that far. More detailed studies are needed in the future, as the enterprise characteristics and workers' line contents vary widely.

In addition, this study has some limitations. First, due to the nature of Internet surveys, selection bias was inevitable, even though data for participants in this study were collected using a diverse selection of sex, occupation, and region to minimize participant bias. Second, because the cohort was relatively short-term (3 months), it may not fully reflect the impact of infection control practices on mental health. Third, as the infection control practices are self-reported by the participants, the response may be tainted by subjective evaluation. However, we believe that misinterpretation of the answers is unlikely to occur because the options within the questions describe specific measures. Finally, the implementation status of infection control practices varies greatly depending on enterprise characteristics. Therefore, enterprise characteristics may also be an alternative indicator in terms of disease control practices. In this study, the analysis is adjusted for company size, worker occupation, and educational background. However, the possibility of the effects of unobserved enterprise characteristics cannot be excluded.

## Conclusions

This study found an association between workers' psychological distress and infection control practices in the working place during the COVID-19 pandemic. Infection control practices may have both positive and negative impacts on workers' mental health. Requests to not come to work if not feeling well were shown to improve workers' mental health, whereas infectious disease control practices that lead to reduced income were shown to worsen workers' distress. Follow-up studies may be necessary to clarify the long-term effects on workers' mental health.

## Data availability statement

The raw data supporting the conclusions of this article will be made available by the authors, without undue reservation.

## Ethics statement

The studies involving human participants were reviewed and approved by Ethics Committee of the University of Occupational and Environmental Health, Japan. The patients/participants provided their written informed consent to participate in this study.

## Author contributions

YF was the chairperson of the study group. TI conceived the research questions. TK analyzed the data with YF and drafted the initial manuscript. All authors were designed this research protocol and developed the questionnaire. All authors read the initial manuscript, revised it, and approved the final manuscript.

## Funding

This study was supported and partly funded by the University of Occupational and Environmental Health, Japan; General Incorporated Foundation (Anshin Zaidan) for the Development of Educational Materials on Mental Health Measures for Managers at Small sized Enterprises; Health, Labor and Welfare Sciences Research Grants: Comprehensive Research for Women's Healthcare (H 30 josei ippan 002) and Research for the Establishment of an Occupational Health System in Times of Disaster (H30 roudouippan 007); scholarship donations from Chugai Pharmaceutical Co., Ltd.; the Collabo Health Study Group; and Hitachi Systems, Ltd. The funders were not involved in the study design, collection, analysis, interpretation of data, the writing of this article or the decision to submit it for publication.

## Conflict of interest

The authors declare that the research was conducted in the absence of any commercial or financial relationships that could be construed as a potential conflict of interest.

## Publisher's note

All claims expressed in this article are solely those of the authors and do not necessarily represent those of their affiliated organizations, or those of the publisher, the editors and the reviewers. Any product that may be evaluated in this article, or claim that may be made by its manufacturer, is not guaranteed or endorsed by the publisher.

## References

[1] Hyland P Shevlin M McBride O Murphy J Karatzias T Bentall RP. Anxiety and depression in the Republic of Ireland during the COVID-19 pandemic. Acta Psychiatr Scand. (2020) 142:249–56. 10.1111/acps.13219 32716520

[2] Shi L Lu ZA Que JY Huang XL Liu L Ran MS. Prevalence of and risk factors associated with mental health symptoms among the general population in China during the coronavirus disease 2019 pandemic. JAMA Netw Open. (2020) 3:e2014053. 10.1001/jamanetworkopen.2020.14053 32609353 PMC7330717

[3] Yao H Chen JH Xu YF. Patients with mental health disorders in the COVID-19 epidemic. Lancet Psychiatry. (2020) 7:e21. 10.1016/S2215-0366(20)30090-0 32199510 PMC7269717

[4] Niedzwiedz CL Green MJ Benzeval M Campbell D Craig P Demou E. Mental health and health behaviours before and during the initial phase of the COVID-19 lockdown: longitudinal analyses of the UK Household Longitudinal Study. J Epidemiol Commun Health. (2021) 75:224–31. 10.1101/2020.06.21.20136820 32978210 PMC7892383

[5] Gursoy D Chi CG. Effects of COVID-19 pandemic on hospitality industry: review of the current situations and a research agenda. J Hosp Mark Manag. (2020) 29:527–9. 10.1080/19368623.2020.1788231

[6] Alon TM Doepke M Olmstead-Rumsey J Tertilt M. The impact of COVID-19 on gender equality [working paper]. NBER. (2020) 26947:1–37. 10.3386/w26947

[7] Dwivedi YK Hughes DL Coombs C Constantiou I Duan Y Edwards JS. Impact of COVID-19 pandemic on information management research and practice: transforming education, work and life. Int J Inf Manag. (2020) 55:102211. 10.1016/j.ijinfomgt.2020.102211

[8] Havaei F Ma A Staempfli S MacPhee M. Nurses' workplace conditions impacting their mental health during COVID-19: a cross-sectional survey study. Healthcare (Basel). (2021) 9:84. 10.3390/healthcare9010084 33467080 PMC7830057

[9] Cho M Kim O Pang Y Kim B Jeong H Lee J. Factors affecting frontline Korean nurses' mental health during the COVID-19 pandemic. Int Nurs Rev. (2021) 68:256–65. 10.1111/inr.12679 33894067 PMC8251381

[10] Benke C Autenrieth LK Asselmann E Pané-Farré CA. Lockdown, quarantine measures, and social distancing: associations with depression, anxiety and distress at the beginning of the COVID-19 pandemic among adults from Germany. Psychiatry Res. (2020) 293:113462. 10.1016/j.psychres.2020.113462 32987222 PMC7500345

[11] Giorgi G Lecca LI Alessio F Finstad GL Bondanini G Lulli LG. COVID-19-related mental health effects in the workplace: a narrative review. Int J Environ Res Public Health. (2020) 17:7857. 10.3390/ijerph17217857 33120930 PMC7663773

[12] Kikuchi H Machida M Nakamura I Saito R Odagiri Y Kojima T. Changes in psychological distress during the COVID-19 pandemic in Japan: a longitudinal study. J Epidemiol. (2020) 30:522–8. 10.2188/jea.JE20200271 32963212 PMC7557175

[13] Yoshioka T Okubo R Tabuchi T Odani S Shinozaki T Tsugawa Y. Factors associated with serious psychological distress during the COVID-19 pandemic in Japan: a nationwide cross-sectional internet-based study. BMJ Open. (2021) 11:e051115. 10.1136/bmjopen-2021-051115 34226236 PMC8260284

[14] Fujino Y Ishimaru T Eguchi H Tsuji M Tateishi S Ogami A. Protocol for a nationwide Internet-based health survey in workers during the COVID-19 pandemic in 2020. J UOEH. (2021) 43:217–25. 10.7888/juoeh.43.217 34092766

[15] Kessler RC Andrews G Colpe LJ Hiripi E Mroczek DK Normand SL. Short screening scales to monitor population prevalences and trends in non-specific psychological distress. Psychol Med. (2002) 32:959–76. 10.1017/S0033291702006074 12214795

[16] Furukawa TA Kawakami N Saitoh M Ono Y Nakane Y Nakamura Y. The performance of the Japanese version of the K6 and K10 in the world mental health survey Japan. Int J Methods Psychiatr Res. (2008) 17:152–8. 10.1002/mpr.257 18763695 PMC6878390

[17] Sakurai K Nishi A Kondo K Yanagida K Kawakami N. Screening performance of K6/K10 and other screening instruments for mood and anxiety disorders in Japan. Psychiatry Clin Neurosci. (2011) 65:434–41. 10.1111/j.1440-1819.2011.02236.x 21851452

[18] Takashi M. Presenteeism: research history and future tasks. Occup Health Rev. (2020) 33:25. 10.34354/ohpfrev.33.1_25

[19] Rosander M. Mental health problems as a risk factor for workplace bullying: the protective effect of a well-functioning organization. Ann Work Expo Health. (2021) 65:1096–106. 10.1093/annweh/wxab040 34145873 PMC8577230

[20] Conway PM Hogh A Rugulies R Hansen ÅM. Is sickness presenteeism a risk factor for depression? A Danish 2-year follow-up study. J Occup Environ Med. (2014) 56:595–603. 10.1097/JOM.0000000000000177 24854252

[21] Dollard MF Tuckey MR Dormann C. Psychosocial safety climate moderates the job demand-resource interaction in predicting workgroup distress. Accid Anal Prev. (2012) 45:694–704. 10.1016/j.aap.2011.09.042 22269559

[22] Erkutlu H Chafra J. Leader psychopathy and organizational deviance: the mediating role of psychological safety and the moderating role of moral disengagement. Int J Workplace Health Manag. (2019) 12:197–213. 10.1108/IJWHM-12-2018-0154

[23] Frazier ML Fainshmidt S Klinger RL Pezeshkan A Vracheva V. Psychological safety: a meta-analytic review and extension. Pers Psychol. (2017) 70:113–65. 10.1111/peps.12183

[24] Ma Y Faraz NA Ahmed F Iqbal MK Saeed U Mughal MF. Curbing nurses' burnout during COVID-19: the roles of servant leadership and psychological safety. J Nurs Manag. (2021) 29:2383–91. 10.1111/jonm.13414 34259372 PMC8420609

[25] Dawel A Shou Y Smithson M Cherbuin N Banfield M Calear AL. The effect of COVID-19 on mental health and wellbeing in a representative sample of Australian adults. Front Psychiatry. (2020) 11:579985. 10.3389/fpsyt.2020.579985 33132940 PMC7573356

[26] Ministry of Internal Affairs Communications. Employment Status Survey. (2018). Available online at: https://www.stat.go.jp/data/shugyou/2017/index.html (accessed July 17, 2021).

[27] Ministry Ministry of Health Labour Welfare. Basic Survey on Wage Structure; 2020. (2021). Available online at: https://www.mhlw.go.jp/toukei/itiran/roudou/chingin/kouzou/z2020/index.html (accessed July 17, 2021).

